# Lateral resolution limit of laser Doppler vibrometer microscopes for the measurement of surface acoustic waves

**DOI:** 10.1038/s41598-021-96684-y

**Published:** 2021-09-07

**Authors:** Robert Kowarsch, Christian Rembe

**Affiliations:** Institute of Electrical Information Technology, Clausthal University of Technology, Leibnizstrasse 28, 38678 Clausthal-Zellerfeld, Germany

**Keywords:** Applied optics, Optical techniques

## Abstract

The lateral or transverse resolution of single-point interferometers for vibration measurement is especially critical for microelectromechanical systems (MEMS) vibrating up to the gigahertz range. In this regime, the acoustic wavelengths are typically in the range of the size of the laser focus. Thus, a successful vibration measurement requires distinct knowledge about the lateral resolution limit and its dependencies with instrumentation parameters. In this paper, we derive an analytic approximation formula, which allows for estimation of the systematic measurement deviation of the vibration amplitude and, thus, a definition of the lateral resolution limit of single-point interferometers for vibration measurement. Further, a compensation and an optimum numerical aperture are proposed the reduce the measurement deviation. For this, the model includes a laser-interferometer microscope of Mach-Zehnder type with Gaussian laser beams considering the Gouy effect and wavefront curvature. As a measurement scenario, an unidirectional surface acoustic wave (SAW) is regarded. The theoretic findings have been validated in the experiment with a representative vibration measurement on a SAW filter at $433\,{\mathrm {MHz}}$ with our heterodyne laser-Doppler interferometer with offset-locked semiconductor lasers. The provided formulas help instrument designers and users to choose suitable instrument parameters, especially the numerical aperture of the utilized microscope objective.

## Introduction

With the advent of the “Internet of Things” (IoT), the interconnection between an abundance of devices (“things”) requires an efficient exploitation of resources for communication, which is formulated in the 5G mobile standard^1,2^. The power-efficient and spectral-selective filtering of the communication channels mainly relies on microacoustic filters up to 6 GHz^3^. In these devices, the electric RF signal is converted to a microacoustic waves, typically surface acoustic waves (SAW) or bulk acoustic waves (BAW). The distinct setup and the resulting microacoustic-wave propagation in these filters defines their spectral-selection characteristics, which are superior to conventional, electric filters in the sub-6 GHz regime^1^. The quality assurance in development and high-volume production of these microacoustic filters require measuring instrumentation which is capable to resolve these microacoustic vibrations with sub-picometer amplitudes at GHz frequencies both spatially and temporally^4^. Heterodyne laser interferometry, also denoted as laser-Doppler vibrometry, is an established technique for the field of microelectromechanical systems (MEMS) testing since it is capable of measuring vibrations contactless and sensitive with femtometer amplitude resolution^5,6^ and a defined uncertainty budget^7^.

One challenge for this instrumentation is to achieve heterodyning techniques capable of measuring vibrations unambiguously at several gigahertz. We, therefore, have proposed heterodyning via two semiconductor lasers which are frequency-offset locked by an optical phase-locked loop^8^. This allows to achieve sub-picometer amplitude resolution for vibrations at several GHz only limited by (half of) the bandwidth of commercially available photodetectors with a flat frequency response. To enable measurements in the GHz regime, several techniques have been proposed, e.g. single-sideband detection^9,10^, pulsed lasers^11–13^ or numerous other techniques^14–16^. The alternative homodyne laser interferometry measures in the baseband and, thus, advantageously exploits the full bandwidth of the photodetectors for the vibration measurement and provides a lower shot-noise limited noise by a factor $1/\sqrt{2}$^17^. As disadvantages, these instruments require a stabilization unless quadrature detection is introduced^17^. Further, these interferometers are prone to non-linearities in the signal-processing chain^18^ and lacks the definition of a defined uncertainty budget^7^. Hitherto, numerous vibration measurements at GHz frequencies have been demonstrated^19–23^.

In most of these publications, the challenge of sufficient spatial resolution of a micro-acoustic waveform with a laser-Doppler vibrometer (LDV) is barely addressed, albeit it is very crucial for the vibration measurement. As a first rough estimation, the size $\Delta x$ of the focused beam must be significantly smaller than the half (acoustic) wavelength $\Lambda$ (${\Delta x < \Lambda /2}$) applying the spatial-sampling interpretation of the Nyquist theorem. However, diffraction limits this beam size (full-width half-maximum, FWHM) depending on the laser wavelength $\lambda$ and the numerical aperture ${\mathrm {NA}}$ of the microscope objective according to Abbe^24^ to

1 $$\begin{aligned} \Delta x_{\mathrm {FWHM}} \approx 0.5\, \frac{\lambda }{\mathrm {NA}} . \end{aligned}$$

Thus, the instrument designer is left with the requirement of ${ \Lambda /\lambda > {\mathrm {NA}}^{-1} }$, which becomes critical at gigahertz frequencies as the acoustic wavelength $\Lambda$ gets in the range of few micrometers. Further, this inequation might misguide to employ the microscope objective with the highest-available numerical aperture.

There are several approaches addressing the lateral resolution for LDV microscopes in a more distinct manner^25,26^. Scruby^25^ has derived a rough approximation for the resolution limit which omits most of the Gaussian beam characteristics. Rembe and Draebenstedt discuss the impact of the Gouy effect of the focused (Gaussian) laser beam for a LDV of Mach–Zehnder-type^26^. The Gouy effect results in a lower phase sensitivity with higher NA (at the waist) and in a non-linear distortion. For small vibration amplitudes and, thus, small phase modulation, this effect can be regarded as a reduced effective wavelength due to the rise in phase propagation velocity^27^. Considering this effect, the highest-available NA might not the best choice for a suitable instrumentation, which is suggested from Eq. (1). The study^26^ on the impact of the Gouy phase concentrates on a single scatterer, which is not applicable for SAWs. Further, the curvature of the Gaussian laser beam with a movement of the surface out of the ideal waist (the vibration itself) is neglected.

In this publication, the measurement of a SAW with a heterodyne laser-Doppler-vibrometer microscope of Mach–Zehnder type is modeled regarding all aspects of a (fundamental) Gaussian beam (Gouy phase and curvature). Based on this Gaussian beam model, a lateral (spatial) resolution limit is derived. We have validated our findings in a representative experiment with a heterodyne LDV with frequency-offset-locked semiconductor lasers at ${632\,{\mathrm {nm}} }$. Our findings are important for laser-Doppler vibrometer designs and the evaluation and interpretation of vibration measurements with such instruments.

## Materials and methods

For the definition of the lateral resolution, the photodetector current due to interference in a heterodyne LDV microscope of Mach–Zehnder type is modeled and the measurement of a surface acoustic wave (SAW) on an ideally-reflecting measurement surface is examined.

### Modeling an interferometer microscope of Mach–Zehnder type

We have modeled the beam propagation, the reflection at the SAW and the interference with Gaussian beams. Thus, a Gaussian beam (measurement beam) propagating in *z* direction impinges on the specimen and is expressed with a (complex) electrical field distribution as^24^

2 $$\begin{aligned} E_{\mathrm {m}} \left( \rho ,z,t \right) \approx E_{\mathrm {m}}^{\mathrm {W}} ( \rho ) \, \exp \left[ { {\mathrm {j}} \, \left( { \omega _{\mathrm {m}} t - \varphi \left( \rho , z \right) + \varphi _0 }\right) } \right] \end{aligned}$$

with the angular frequency $\omega$, the phase $\varphi$, a constant phase $\varphi _0$ and ${{\mathrm {j}} = \sqrt{-1} }$. Here, for axially-symmetric functions, a cylindrical coordinate system is preferred with the radius $\rho$ and the axial coordinate *z*. Otherwise, a Cartesian coordinate system is used with the lateral coordinates *x* and *y* (${ \rho ^2 = x^2 + y^2 }$). The use of the Gaussian beam model implicates that all field components in *z* direction are neglectable (low wavefront curvatures) and, thus, only moderate numerical apertures are modeled properly. In this paraxial approximation, the tangents of the beam divergence is approximately equal to the numerical aperture: ${\tan \theta \approx \sin \theta = {\mathrm {NA}} }$. This approximation is applicable for ${{\mathrm {NA}} < 0.6}$ when a relative deviation of less than 20% is tolerated. Further, it should be noted that a Gaussian beam has always to be regarded an approximation of real scenario since this model would require infinite aperture sizes, which do not exist. As a rule of thumb, any aperture diameter in the beam path must be 50% larger than the local beam $1/e^2$-diameter, so that diffraction effects can be neglected.

The Gaussian spot profile $E^{{\mathrm {W}}}$ at the waist has the field distribution

3 $$\begin{aligned} E_{\mathrm {m}}^{\mathrm {W}} (\rho ) \approx \hat{E}_{\mathrm {m}} \, \exp \left( - \frac{\rho ^2}{w_0^2} \right) \end{aligned}$$

with the amplitude $\hat{E}_{\mathrm {m}}$ of the electromagnetic (EM) wave impinging on the specimen. This fundamental Gaussian beam profile is also considered as ${{\mathrm {TEM}}_{00}}$ in the nomenclature of Hermite–Gaussian modes^24^.

As the angular frequency $\omega _{\mathrm {m}}$ of an (optical) EM wave is beyond the bandwidth of conventional photodetectors, interference with a reference EM wave at the angular frequency $\omega _{\mathrm {r}}$ is incorporated (coherent detection). Therefore, on the photodetector, the EM wave $E_{\mathrm {m}}$ of the measurement beam is interfered with the reference beam $E_{\mathrm {r}}$. The photodetector integrates the intensity over its sensitive area $A_{\mathrm {D}}$ (orthogonal to the *z* axis) and at the photodetector position $z_{\mathrm {D}}$ the interference generates the current signal

4 $$\begin{aligned} i_{\mathrm {D}} \left( t \right)&= {S_{\mathrm {D}}} \, \frac{c\,{\epsilon _0}}{2} \iint \limits _{A_{\mathrm {D}}} {{\left| {{E_{\mathrm {m}}\left( \rho ', z_{\mathrm {D}}, t \right) } + {E_{\mathrm {r}}}\left( \rho ', z_{\mathrm {D}}, t \right) } \right| }^2} \, {\mathrm {d}}A' \nonumber \\&= {S_{\mathrm {D}}}\, \frac{c\,{\epsilon _0}}{2} \iint \limits _{A_{\mathrm {D}}} {\left| {E_{\mathrm {m}}\left( \rho ', z_{\mathrm {D}}, t \right) } \right| }^2 + {\left| {E_{\mathrm {r}}\left( \rho ', z_{\mathrm {D}}, t \right) } \right| }^2 + 2 \, {\mathfrak {R}}\left\{ { {E^*_{\mathrm {m}}}\left( \rho ', z_{\mathrm {D}}, t \right) \, {E_{\mathrm {r}}}\left( \rho ', z_{\mathrm {D}}, t \right) }\right\} \, {\mathrm {d}}A' \end{aligned}$$

with the electric constant ${\epsilon _0}$. The photodetector sensitivity ${S_{\mathrm {D}}}$ (in ${\mathrm {A/W}}$) is assumed to be constant over the sensitive area $A_{\mathrm {D}}$ of the photodetector. The local coordinate system is denoted with a dash, e.g. the radial coordinate $\rho '$ and the area element ${\mathrm {d}}A'$ in the local coordinate system on the photodetector. The asterisk means the complex conjugation and ${\mathfrak {R}}\left\{ \cdot \right\}$ the real part.

The first two terms in Eq. (4) describe the incoherent superposition of the interfering EM waves and the last term the coherent interference. It is assumed that the propagation paths of the interfering EM waves are chosen that the interference (on the photodetector) occurs within the coherence length of a suitable laser source. As the DC part is of minor interest here, it is omitted in the following. Technically, this is achieved by AC coupling of the current signal after the photodetection.

**Figure 1:**
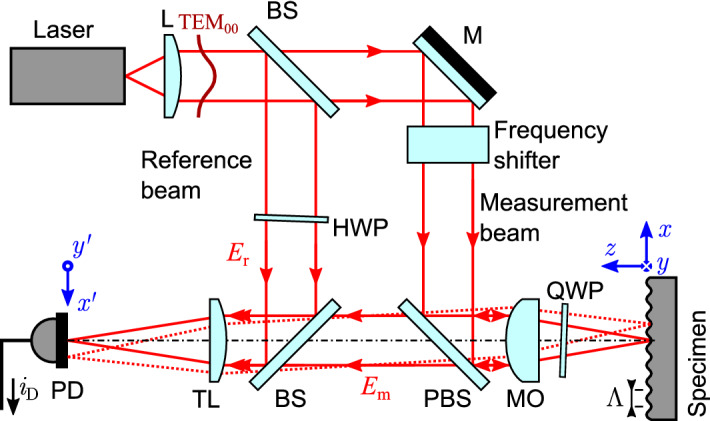
Schematic setup of the heterodyne LDV microscope of Mach-Zehnder type for the measurement of a surface acoustic wave (with an acoustic wavelength $\Lambda$). The wavefront on the specimen (coordinates *x* and *y*) is imaged onto the photodetector with the local coordinate system ($x',\,y'$). BS = beam splitter, M = mirror, L = lens, TL = tube lens, PBS = polarizing beam splitter, MO = microscope objective, QWP = quarter-wave plate, HWP = half-wave plate, PD = photodetector.

In a LDV microscope of Mach–Zehnder type (Fig. 1), the measurement beam is focused with a microscope objective onto the specimen. The scattered radiation is collected by the same objective and interferes with the reference beam on the photodetector. To achieve high frequencies, the sensitive area of a photodetector must be small for a low electric capacitance. Thus, it is assumed that both beams are focused (with a tube lens) onto the photodetector and the photodetector is located in the waist of both (Gaussian) beams. For this, the wavefront on the specimen is imaged with the magnification $\beta$ onto the photodetector. The magnification $\beta$ is directly dependent on the focal-length ratio of the microscope objective and the tube lens ^24^.

For the derivation of a lateral resolution a plane surface acoustic wave (SAW) on the homogeneous-reflective specimen (traveling in *x* direction) is assumed with

5 $$\begin{aligned} s(x,t) = - \hat{s} \, {\sin \left( { {\omega _{{{\mathrm {vib}}}}}t - \frac{2\pi }{\Lambda } x } \right) } \, . \end{aligned}$$

The acoustic wavelength $\Lambda$ of the surface acoustic wave (SAW) and the vibration frequency ${ f_{\mathrm {vib}} = \omega _{\mathrm {vib}}/2\pi }$ are linked via the acoustic propagation speed $c_{\mathrm {ac}}$ with

6 $$\begin{aligned} \Lambda = \frac{c_{{\mathrm {ac}}}}{f _{{\mathrm {vib}}}} . \end{aligned}$$

Each complex microacoustic (out-of-plane) waveform can be decomposed (after Fourier) in a superposition of harmonic SAWs at different frequencies. Thus, from this simple case, one can also conclude to standing acoustic waves or even complex acoustic waveforms. For the simplicity it is assumed that the surface normal is collinear with the axial coordinate *z* of the impinging laser beam.

### Phase at the waist of the Gaussian beam

The total phase argument of a Gaussian beam with the field in Eq. (2) is ^24^

7 $$\begin{aligned} \varphi \left( \rho , z \right) = \frac{2\pi }{\lambda }\,z + \frac{2\pi }{\lambda }\,\frac{ \rho ^2}{2\,R\left( z\right) } - \xi \left( z \right) \end{aligned}$$

with the wavelength $\lambda$. The radius of curvature is

8 $$\begin{aligned} R \left( z \right) = z \left[ {1 + {{\left( {\frac{{{z_{\mathrm {R}} }}}{z}} \right) }^2}} \right] \; {\mathop \approx \limits ^{ \mathrm {waist} }} \; {\frac{{{z_{\mathrm {R}}^2 }}}{z}} \end{aligned}$$

and the Rayleigh range (using the beam parameter product of a Gaussian beam^24^)

9 $$\begin{aligned} z_{\mathrm {R}} = \frac{\pi }{\lambda }\,w_0^2 = \frac{\lambda }{\pi } \tan ^2 \left( { \theta _{\mathrm {div}} }\right) \end{aligned}$$

with waist radius $w_0$ and the divergence angle $\theta _{\mathrm {div}}$. The Gouy effect results in an additional phase

10 $$\begin{aligned} \xi \left( z \right) = {\arctan \left( {\frac{z}{z_{{\mathrm {R}}}}} \right) } \; {\mathop \approx \limits ^{ \mathrm {waist} }} \; {\frac{z}{z_{{\mathrm {R}}}}}. \end{aligned}$$

If only small amplitudes (${\hat{s} \ll \lambda }$) are expected and the specimen is located in the waist of the beam, the effects on to the total phase can be linearized. Therefore, the linearized dependency of the phase $\varphi$ from the displacement *z* at the waist (${z \approx 0}$) is

11 $$\begin{aligned} \varphi \left( \rho , z \right) \approx \frac{2\pi }{\lambda }\,z +\frac{2\pi }{\lambda } \, \frac{ \rho ^2 }{ 2 \, z^2_{\mathrm {R}}} \, z -{\frac{z}{z_{{\mathrm {R}}}}} = \frac{2\pi }{\lambda } \left( 1 + \frac{ \rho ^2 }{2\,z^2_{\mathrm {R}}} - \frac{\lambda }{2\pi } \, \frac{1}{z_{{\mathrm {R}}}} \right) \, z = \frac{2\pi }{\lambda } \, \Phi \left( { \rho }\right) \,z \end{aligned}$$

with the phase factor

12 $$\begin{aligned} \Phi \left( { \rho }\right) = 1 + \frac{\rho ^2}{2\,z^2_{\mathrm {R}}} - \frac{\lambda }{2\pi } \, \frac{1}{z_{{\mathrm {R}}}} \end{aligned}$$

which includes the contributions both of curvature of the wavefront (2nd term) and of the Gouy effect (3rd term).

### Modeling the resulting photodetector signal

The AC photocurrent signal results from the interference term in Eq. (4) which is dependent on the phase difference between the interfering EM waves on the photodetector. The wavefront of the measurement beam is modulated by the SAW both in *x* direction and time. Further, due to the reflection-microscope setup, the amplitude of phase modulation is doubled. The wavefront of the reference beams remains collimated and ideally flat for the Mach–Zehnder setup (Fig. 1). It should be noted, that this is also applicable to LDV setups of Linnik- or Mireau-type ^28^. Hence, the phase difference of the interference is the phase modulation due to the SAW on the specimen, which is imaged onto the photodetector. Further, with heterodyning, an (angular) frequency offset ${\omega _{\mathrm {c}} = |\omega _{\mathrm {m}} - \omega _{\mathrm {r}}|}$ is introduced which is denoted as (heterodyne) carrier frequency. Other (differential) phase fluctuations of the laser sources are neglected here (find a more extensive discussion in our further publication^8^).

With the approximated phase (at the waist) from Eq. (11), this results in the AC current signal on the photodetector

13 $$\begin{aligned} i_{\mathrm {D}} \left( { t }\right)&\propto \iint \limits _{A_{{\mathrm {D}}}} { E_{{\mathrm {m}}}^{{\mathrm {W}}} \left( { \rho ' }\right) \, E_{{\mathrm {r}}}^{{\mathrm {W}}} \left( { \rho ' }\right) \, {\mathfrak {R}}\left\{ {\exp \left[ {\mathrm {j}} \, { {\omega _{{\mathrm {c}}}} t } -{\mathrm {j}} \, \frac{2\pi }{\lambda }\Phi \left( \rho ' \right) \,2 s \left( { x , t }\right) + {\mathrm {j}}\,\varphi _0 \right] } \right\} } {\mathrm {d}}A' \end{aligned}$$

with the constant phase $\varphi _0$ due to the static propagation (difference) of both beams within the interferometer. With the SAW from Eq. (5), the AC current signal with the substitution of ${ x' = \beta \, x }$ and ${ \rho ' = \beta \, \rho }$ , which models the imaging with the magnification $\beta$, becomes

14 $$\begin{aligned} i_{\mathrm {D}} \left( { t }\right)&\propto {\mathfrak {R}}\biggl \{ \iint \limits _{A_{{\mathrm {D}}}} E_{{\mathrm {m}}}^{{\mathrm {W}}} \left( { \rho ' }\right) \, E_{{\mathrm {r}}}^{{\mathrm {W}}} \left( { \rho ' }\right) \, {\exp \left[ {\mathrm {j}} \left( {{\omega _{{\mathrm {c}}}} t + \varphi _{0} } \right) \right] } \; {\exp \left[ {\mathrm {j}} \, \frac{{4\pi \, \Phi \left( { \rho ' }\right) }}{\lambda } \, \hat{s}\, \sin \left( { \omega _{{\mathrm {vib}}} t - 2\pi \frac{x'}{\Lambda \,\beta } } \right) \right] } \,{\mathrm {d}}A' \biggr \} . \end{aligned}$$

The approximation for small vibration amplitudes (${\hat{s} \ll \lambda /2}$) is shown in more detail in the supplementary information. Thereafter, the AC current from equation  (SI.2) is

15 $$\begin{aligned} i_{\mathrm {D}} \left( { t }\right)&\propto {{\mathfrak {R}}} \biggl \{ \iint \limits _{A_{{\mathrm {D}}}} E_{{\mathrm {m}}}^{{\mathrm {W}}} \left( { \rho ' }\right) \, E_{{\mathrm {r}}}^{{\mathrm {W}}} \left( { \rho ' }\right) \, \exp \left[ { {\mathrm {j}} \, \left( { {\omega _{{\mathrm {c}}}} \, t +\varphi _0} \right) } \right] \left[ {1 \pm \frac{{2\pi \, \Phi \left( { \rho ' }\right) }}{\lambda } \, \hat{s} \, \exp \left( \pm {\mathrm {j}}\, {\omega _{{\mathrm {vib}}} t \mp {\mathrm {j}}\,2\pi \frac{x'}{\Lambda \,\beta } } \right) }\right] \, {\mathrm {d}}A' \biggr \} . \end{aligned}$$

Thus, the resulting AC current signal can be described with

16 $$\begin{aligned} i_{\mathrm {D}} \left( { t }\right) \approx {{\mathfrak {R}}} \left\{ { {{\hat{i}}_{{\mathrm {c}}}}\, \exp \left[ { {\mathrm {j}} \, \left( {{\omega _{{\mathrm {c}}}} t + \varphi _0 } \right) } \right] } \pm {{{\hat{i}}_{\mathrm {J}1}} \, \exp \left[ { {\mathrm {j}} \, \left( {{\omega _{\mathrm {c}}} t \pm {\omega _{\mathrm {vib}}} t + \varphi _0 } \right) } \right] } \right\} \end{aligned}$$

with the (real) carrier current amplitude

17 $$\begin{aligned} {{\hat{i}}_{\mathrm {c}}} = {S_{\mathrm {D}}} \, \frac{c\,{\epsilon _0}}{2} \iint \limits _{A_{\mathrm {D}}} E_{\mathrm {m}}^{\mathrm {W}} \left( { \rho ' }\right) \, E_{\mathrm {r}}^{\mathrm {W}} \left( { \rho ' }\right) \, {\mathrm {d}}A' \end{aligned}$$

and with the current amplitude of the first sideband

18 $$\begin{aligned} {{\hat{i}}_{{\mathrm {J}}1}} = {S_{\mathrm {D}}} \, \frac{c\,{\epsilon _0}}{2} \, {{\mathfrak {R}}} \left\{ \iint \limits _{A_{{\mathrm {D}}}} E_{\mathrm {m}}^{\mathrm {W}} \left( { \rho ' }\right) \, E_{\mathrm {r}}^{\mathrm {W}} \left( { \rho ' }\right) \, { { { \frac{{2\pi \, \Phi \left( { \rho ' }\right) }}{\lambda } \, \hat{s} \, \exp \left( \pm {\mathrm {j}}\, {\omega _{\mathrm {vib}} t \mp {\mathrm {j}}\,2\pi \frac{x'}{\Lambda \,\beta } } \right) } }}\, {\mathrm {d}}A' \right\} . \end{aligned}$$

It is to be noted, that the frequency offset $\omega _{\mathrm {c}}$ for homodyne interferometry is zero and, thus, the DC components are not separable from the “carrier” signal. Nevertheless, the resulting systematic effects on the measurement are transferable.

### Systematic vibration-amplitude deviation after reconstruction

The vibration amplitude can be calculated^6^ (for ${ \hat{s} \ll \lambda /2 }$) from the ratio of the current amplitude at the sideband and at the carrier with

19 $$\begin{aligned} {\hat{s}_{\mathrm {est}}} \approx \frac{\lambda }{2\pi } \, \frac{{\hat{i}}_{\mathrm {J}1}}{{\hat{i}}_{\mathrm {c}}} . \end{aligned}$$

Thus, the ratio of the reconstructed vibration amplitude ${\hat{s}_{\mathrm {est}}}$ and the actual vibration amplitude ${\hat{s}}$ is

20 $$\begin{aligned} {\frac{{{\hat{s}}_{{\mathrm {est}}}}}{\hat{s}}} \approx {\frac{\displaystyle {\mathfrak {R}}\left\{ {\iint \limits _{{A_{\mathrm {D}}}} { E_{\mathrm {m}}^{\mathrm {W}} \left( { \rho ' }\right) \, E_{\mathrm {r}}^{\mathrm {W}} \left( { \rho ' }\right) \, \Phi \left( \rho ' \right) \, \exp \left( {- {\mathrm {j}} \, 2\pi {\frac{x'}{\Lambda \,\beta }}} \right) \, {\mathrm {d}}{A'}} } \right\} }{\displaystyle {\iint \limits _{{A_{\mathrm {D}}}} { E_{\mathrm {m}}^{\mathrm {W}} \left( { \rho ' }\right) \, E_{\mathrm {r}}^{\mathrm {W}} \left( { \rho ' }\right) \, \, {\mathrm {d}}{A'} } } } } . \end{aligned}$$

Hence, a ratio ${ {{{{\hat{s}}_{{\mathrm {est}}}}} / {\hat{s}}} = 1 }$ is desirable, meaning an ideal reconstruction of the vibration amplitude. The analytic solution of the integrals is shown in full detail in the supplementary information. The solution yields an approximation formula which is only dependent on the numerical aperture ${\mathrm {NA}}$ of the microscope objective or the beam waist radius $w_{0}$ and the wavelength ratio $\Lambda /\lambda$ of the acoustic wavelength $\Lambda$ to the laser wavelength $\lambda$

21 $$\begin{aligned} {\frac{{{\hat{s}}_{{\mathrm {est}}}}}{\hat{s}}}&\approx {{ \left( 1 - \frac{{\mathrm {NA}}^2 }{4} - \frac{\lambda ^2}{8 \, \Lambda ^2 } \right) \exp \left( { - \frac{{\lambda ^2}}{2 \, \Lambda ^2} \, \frac{1}{{\mathrm {NA}}^2} } \right) } } . \end{aligned}$$

The dependency of ${\mathrm {NA}}$ (of the Gaussian beam) gaslights to directly insert the NA of the microscope objective. This would however lead to diffraction, since the (entrance) pupil diameter would be in the range of the diameter of the laser beam. Consequently, a deviation from the deduced behavior in Eq. (21) must be expected. Thus, as a rule of thumb, the pupil diameter must be 50% larger than the local (Gaussian) beam diameter.

**Figure 2:**
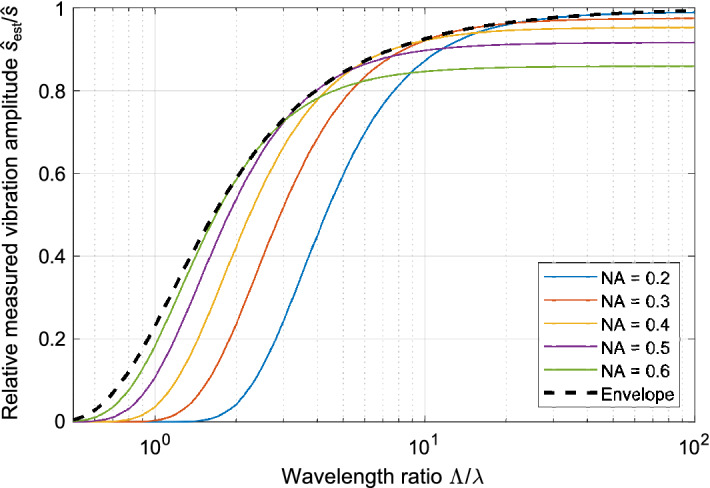
Ratio of measured to real vibration amplitude ${{{{\hat{s}}_{{\mathrm {est}}}}} /{\hat{s}}}$ dependent on the wavelength ratio $\Lambda /\lambda$ for several numerical apertures NA according to Eq. (22). The set of curves shows an envelope which defines the smallest-achievable systematic deviation.

From the Eq. (21), the relative vibration amplitude ${{{{\hat{s}}_{{\mathrm {est}}}}} /{\hat{s}}}$ measured with the LDV microscope is directly related to the wavelength ratio ${\Lambda / \lambda }$. For a physically realizable value (${{{{\hat{s}}_{{\mathrm {est}}}}} /{\hat{s}}>0}$), this equation yields a hard boundary of ${\Lambda >\lambda /8}$, which is beyond practical use. Thus, the influence of this term can be neglected yielding

22 $$\begin{aligned} {\frac{{{\hat{s}}_{{\mathrm {est}}}}}{\hat{s}}}&\approx {{ \left( 1 - \frac{{\mathrm {NA}}^2 }{4} \right) \exp \left( { - \frac{{\lambda ^2}}{2 \, \Lambda ^2} \, \frac{1}{{\mathrm {NA}}^2} } \right) } } \end{aligned}$$

and is shown in Fig. 2 for several NAs.

The second factor ${\mathrm {NA}}^2/4$ in Eq. (22) shows the combination of the Gouy effect and the effect of wavefront curvature which oppose each other. The Gouy effect decreases phase velocity, whereas the wavefront curvature increases the phase velocity (off the center). This can be understood by analyzing the two numerator integrals ${\Upsilon _1}$ and ${\Upsilon _2}$ in the analytic solution in the supplementary information.

For large wavelength ratios ${\Lambda /\lambda }$, Eq. (22) converges to the well-known correction formula from Ingelstam for step heights in interferoemtric topography metrology^29^. For small numerical apertures (${\mathrm {NA}} < 0.16$), the curvature near the waist and the Gouy effect might be neglected (deviation of phase factor $\Phi$ in Eq. (12) less than $10\%$). Hence, Eq. (22) can be approximated with

23 $$\begin{aligned} \frac{{\hat{s}}_{\mathrm {est}}}{\hat{s}} \approx \exp \left( { - \frac{{\lambda ^2}}{2\, \Lambda ^2} \, \frac{1}{{\mathrm {NA}}^2} } \right) \approx \exp \left( { - \frac{{\pi ^2 \, w_{0}^2}}{2 \, \Lambda ^2} } \right) \end{aligned}$$

utilizing Eq. (9). With a Taylor expansion for a small laser focus compared to the acoustic wavelength (${w_0 < \Lambda }$), this is consistent with the approximation from Scruby^25^ for the LDV measurement of harmonic surface modulation.

Thus, the derived approximation for LDV microscopy in Eq. (22) describes a combination of Ingelstam’s and Scruby’s approximations^25,29^.

## Results and discussion

### Lateral resolution limit

For high numerical apertures NA, the Gouy phase and wavefront curvature have to be considered, which is incorporated in Eq. (21). This results in a systematic deviation of the reconstructed vibration amplitude in respect to the real vibration amplitude that can be compensated by multiplication of the measured vibration amplitude with a factor *C* (previously known from Ingelstam ^29^)

24 $$\begin{aligned} C = {\left( {1 - \frac{{\mathrm {NA}}^2}{4} }\right) }^{-1} . \end{aligned}$$

It should be noted, that this factor *C* from the Gouy phase is smaller than shown for a flat wavefront in a previous publication^26^. Other models in literature also overcompensate the effect^30^. From the number of assumptions leading to Eq. (21), it should be noted that there is already a significant uncertainty budget for the compensation of systematic effects. For example, an inhomogeneous surface reflectivity alters the systematic effect and results in a higher uncertainty for the compensation.

Generally, it follows that a small wavelength ratio (${\Lambda /\lambda \le 10}$) results in a systematic deviation of the reconstructed vibration amplitude $\hat{s}_{\mathrm {est}}$ to the real amplitude $\hat{s}$. This results in a limited capability of resolving a SAW with a LDV microscope. Coherent with a previous publication^26^, the lateral resolution limit is defined by demanding that the instrument does not exceed a relative systematic deviation ${\alpha = \left| \hat{s}_{\mathrm {est}} - \hat{s} \right| / \hat{s}}$, e.g. 5%. This approach is applicable for the rough approximations in Eq. (23). Demanding ${\alpha \le 5\%}$, this results in

25 $$\begin{aligned} w_{0} \lesssim \frac{1}{\pi }\, \sqrt{2\,\left| \ln \left( 1-\alpha \right) \right| } \, {\Lambda } \approx 0.10 \, {\Lambda } , \end{aligned}$$

which means that the waist radius $w_{0}$ of a Gaussian beam has to be minimum 10 times smaller than the acoustic wavelength $\Lambda$ of the SAW which is to be measured. Applying the beam parameter product of the Gaussian beam^24^, the minimum-resolvable acoustic wavelength ${ {\Lambda } }$ is

26 $$\begin{aligned} {\Lambda } \gtrsim \frac{1}{ \sqrt{2\,\left| \ln \left( 1-\alpha \right) \right| }} \, \frac{\lambda }{\mathrm {NA}} \approx {3.12} \, \frac{\lambda }{\mathrm {NA}} \end{aligned}$$

The approximation Eq. (26) indicates that for the proper lateral resolution of a SAW, the full-width $\Delta x$ (at half maximum) from Eq. (1) has to more than six times smaller than the minimum acoustic wavelength $\Lambda$. This requirement for the measurement of a SAW on a specimen is, thus, more demanding than the resolution limit for the measurement of adjacent, independently-moving scatterers impaired by three-wave interference^6,26^.

### Impact on the measurement with state-of-the-art LDV microscopes

The following example shows the demand for high lateral resolution for LDV microscopes especially when vibrations in ultra-high-frequency microsystems vibrating at several gigahertz are to be measured. With Eq. (6), the capability to measure a SAW at a frequency $f_{\mathrm {vib}}$ is impaired by the limited lateral resolution according to Eq. (25) which leads to the limitation

27 $$\begin{aligned} f_{\mathrm {vib}} \lesssim 0.32 \,c_{\mathrm {ac}} \, \frac{\mathrm {NA}}{\lambda } . \end{aligned}$$

For example, the acoustic propagation speed for a SAW on Lithium Niobate (${\mathrm {LiNbO_3}}$) is ${{c_{\mathrm {ac}}}} \approx 3960\,{{\mathrm {m}}/{\mathrm {s}}}$ for a typical $128^\circ$ X–Y cut. Further, we assume a LDV microscope, which has a numerical aperture of 0.55 at the laser wavelength ${ \lambda = 532\,{\mathrm {nm}} }$ (assuming an effective NA for the Gaussian beam of ${ {\mathrm {NA}} = 0.37 }$). According to the lateral resolution limit in Eq. (27) (without the Gouy effect), this characteristics result in a capability to measure vibration frequencies of less than ${ 900\,{\mathrm {MHz}} }$ for SAWs (on ${\mathrm {LiNbO_3}}$), unless larger deviations than ${\alpha \le 5\%}$ of the reconstructed vibration amplitude are accepted. Thus, even if such a LDV microscope specifies a signal processing capability with a maximum frequency of more than $2\,{\mathrm {GHz}}$, the frequency range for vibration measurements can be strongly limited by the lateral resolution of the LDV instrument dependent on the specimen.

Therefore, a thoughtful and deliberate choice of the parameters for the LDV measurement is crucial to avoid excessive deviations. Further, the demand for super-resolution techniques also for vibrometry on ultra-high-frequency microsystems in the gigahertz regime becomes obvious. As a promising method based on absorbance modulation is proposed in our further publication^31^.

### Optimization of the numerical aperture

**Figure 3:**
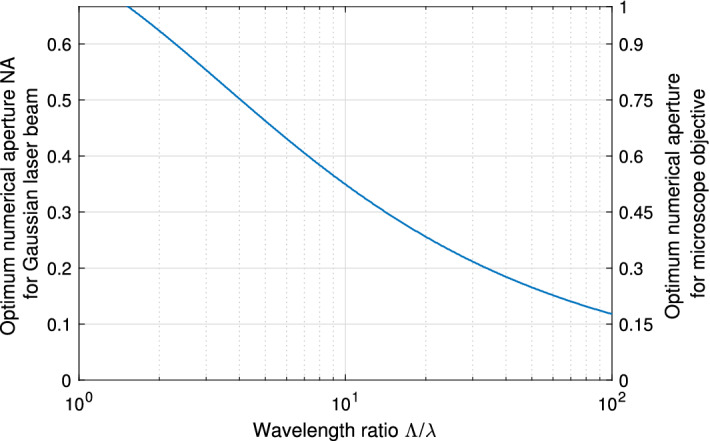
Optimum numerical aperture ${\mathrm {NA_{opt}}}$ for a minimum systematic vibration-amplitude deviation in dependency on the wavelength ratio $\Lambda /\lambda$. For the validation, the numerical aperture (NA) of the microscope objective has to exceed the NA of the Gaussian beam (assumed here with 50%), so that neglectable diffraction occurs. Therefore, on the right, a second *y* axis shows the (minimum) NA of the corresponding microscope objective.

An useful behavior (see Fig. 2) is the formation of an envelope (dashed) where the minimum systematic deviation over all NAs for a specific wavelength ratio $\Lambda /\lambda$ is obtained. With this envelope, the optimum numerical aperture ${\mathrm {NA_{opt}}}$ for an expected wavelength ratio $\Lambda /\lambda$ can be identified. The optimum NA for Fig. 3 was found in a numerical optimization with MATLAB (searching the NA at which the ratio ${\hat{s}_{\mathrm {est}}/\hat{s}}$ in approximation Eq. (22) is maximum for a given wavelength ratio $\Lambda /\lambda$). For example, the minimum deviation for the wavelength ratio ${ \Lambda /\lambda = 10 }$ is achieved for an optimum numerical aperture ${\mathrm {NA_{opt}} = 0.35 }$ and, hence, a minimum NA of the microscope objective of 0.53 (vertical axis on the right) to maintain the Gaussian beam (meaning that the local $1/e^2$-diameter at the pupil of the microscope objective has to be $50\%$ larger than the physical aperture). With this finding, a numerical aperture can be deliberately chosen, which results in a minimum deviation of the reconstructed vibration amplitude to the real vibration amplitude.

### Experimental validation

For the experimental validation, we employed our heterodyne LDV, which we describe in detail in a further publication^8^. This LDV achieves high-frequency measurement capability via heterodyning with two frequency-offset-locked semiconductor lasers at ${632\,\mathrm {nm}}$ and broadband photodetectors. One semiconductor laser provides the measurement beam and the second semiconductor laser provides the reference beam of the Mach-Zehnder-type LDV. A microscope objective (Mitutoyo Plan Apo ${50\times }$ with ${\mathrm {NA}} = 0.55$) focuses the measurement beam onto the specimen.

For the experiments, we chose a SAW filter at ${433.35\,{\mathrm {MHz}}}$ (Epcos B3530) and performed a single-point vibration measurement on the central, reflective shielding between the interdigital transducers of the two ports. The acoustic wavelength $\Lambda$ of the SAW filter is ${ 7.3\,\upmu {\mathrm {m}} }$ resulting in a wavelength ratio ${\Lambda /\lambda = 11.5}$ for this experiment. For the variation of the numerical aperture NA, we changed the illumination of the entrance pupil at the microscope objective via an iris. The illuminated diameter of the entrance pupil and, thus, the numerical aperture NA was determined with a camera (Basler acA1920-40ua), see equation  (SI.25) in the supplementary information for conversion. For smaller apertures, a more diffraction-limited spot is generated on the specimen, which can be approximated with a Gaussian spot profile^32^. The resulting wavefront, however, is more deformed in respect to the pure Gaussian mode. This might result in a larger deviation of the measured vibration amplitude than from the Gouy effect (for high numerical apertures).

**Figure 4:**
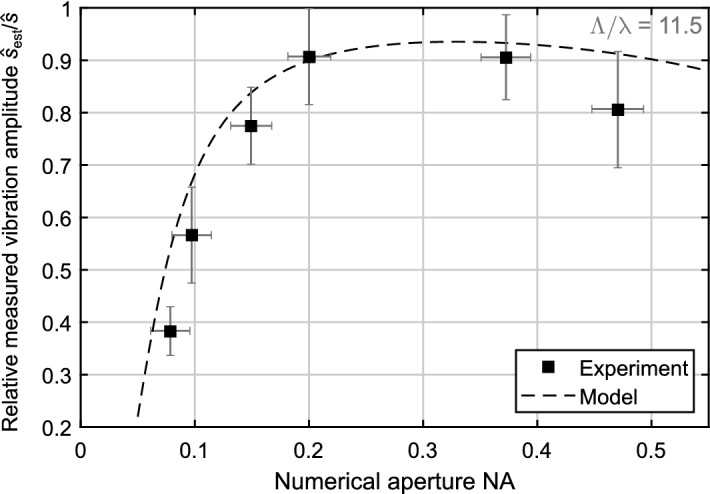
Experimental validation of the approximation Eq. (22) with our heterodyne LDV with frequency-offset-locked semiconductor lasers. Therefore, a vibration measurement was conducted on a SAW filter at ${433.35\,{\mathrm {MHz}}}$. The reduction of the numerical aperture was achieved with a iris at the entrance pupil of a ${50 \times }$ microscope objective with ${ {\mathrm {NA}} = 0.55 }$.

The experimental results are shown in Fig. 4 compared to the approximation Eq. (22). The maximum vibration amplitude (at ${\mathrm {NA} = 0.2}$) was ${\hat{s}_{\mathrm {est}} = 160\,{\mathrm {pm} }}$ with a noise-equivalent vibration amplitude of less than ${ 20\,{\mathrm {pm}} }$. For each numerical aperture, ten vibration measurements were conducted and complex-averaged (with the excitation signal as phase reference). The real vibration amplitude was determined with a least-mean-squares fit of the approximation Eq. (22) to the data points of the experiment with $\hat{s} = 175\,{\mathrm {pm}}$. For all measured values, the uncertainty was determined and is shown as error bars in Fig. 4 (further explanation in supplementary information, confidence level $68.3\%$).

The dependency of the relative measured vibration amplitude ${\hat{s}_{\mathrm {est}} / \hat{s}}$ from the numerical aperture NA follows the approximation Eq. (22). The remaining deviation between experiment and approximation equation is within the boundaries of the measurement uncertainties. However, residual in-plane vibrations, drift of the positioning stage and deviations from the pure Gaussian beam model, e.g. aberrations, might also cause deviations. Especially, the significant stronger decay for higher numerical apertures might be due to the more-deformed wavefront of measurement beam compared to a pure Gaussian beam. The reason might be the moderate beam quality of the semiconductor laser and aberrations. This effect may also be responsible for a lower optimum NA compared to the theoretic optimum NA of 0.3 from Fig. 3. Thus, this experiment indicates the validity of our approach with the deduced approximations.

## Conclusions

This publication gives a theoretic estimation of the lateral (spatial) resolution limit of single-point interferometer microscopes for the vibration measurement of surface acoustic waves. With this approach, an approximation formula of the lateral resolution limit on the numerical aperture and the wavelength is deduced. A similar relation is known from the Abbe resolution limit for incoherent microscopy, however the (coherent) vibrometer microscope requires a six times better lateral resolution to obtain a vibration measurement with neglectable measurement deviations. Considering the Gouy phase and the wavefront curvature of the Gaussian beam model, we deduced an optimum numerical aperture, which minimizes the systematic measurement deviation for a given ratio of the acoustic wavelength to the laser wavelength. Further, a compensation of the residual systematic effect is proposed. Our findings help both designers and users to deliberately choose an appropriate numerical aperture of their vibrometer microscope dependent on the application. In an experiment, the theoretic findings have been validated with a vibration measurement on a SAW filter.

## Supplementary Information

Supplementary Information.
