# Supplementary material for: Lateral resolution limit of laser Doppler vibrometer microscopes for the measurement of surface acoustic waves
Source: Sci Rep. 2021 Sep 7;11:17753. doi: 10.1038/s41598-021-96684-y (PMC8423847; doi:10.1038/s41598-021-96684-y)
Supplement: Supplementary file 1 — Supplementary Information. [file 41598_2021_96684_MOESM1_ESM.pdf]

Supplementary information
Lateral resolution limit of laser Doppler vibrometer microscopes for the measurement of surface acoustic waves

Robert Kowarsch[1,*] and Christian Rembe[1]

[1]Institute of Electrical Information Technology, Clausthal University of Technology, Leibnizstrasse 28, 38678 Clausthal-Zellerfeld, Germany
[*]kowarsch@iei.tu-clausthal.de

1 Approximation for small vibration amplitudes

The simplification of equation (14) in the main document is done by the Jacobi-Anger identity[1]

$$\exp\left(\mathrm{j}\,a\,\sin\varpi\right) = \sum_{m=-\infty}^{\infty} \mathrm{J}_m(a)\,\exp\left(\mathrm{j}\,m\,\varpi\right) \tag{SI.1}$$

with the Bessel function J_m of first kind and the order J_m ($m \in \mathbb{N}$). ϖ is an arbitrary phase argument and a is a coefficient. Thus, the AC current signal from equation (14) in the main document is

$$i_\mathrm{D}(t) \propto \Re\left\{\iint\limits_{A_\mathrm{D}} E_\mathrm{m}^\mathrm{W}(\rho')\,E_\mathrm{r}^\mathrm{W}(\rho')\,\exp\left[\mathrm{j}\left(\omega_\mathrm{c}t+\varphi_0\right)\right]\sum_{m=-\infty}^{\infty}\mathrm{J}_m\left(\frac{4\pi\,\Phi(\rho')}{\lambda_\mathrm{m}}\,\hat{s}\right)\exp\left[\mathrm{j}m\left(\omega_\mathrm{vib}t-2\pi\frac{x'}{\Lambda\beta}\right)\right]\mathrm{d}A'\right\}. \tag{SI.2}$$

Microaoustic vibrations especially at gigahertz frequencies typically have small vibration amplitudes $\hat{s}$ in relation to the laser wavelength λ. This is reasonable since the energy is proportional to the square of the vibration velocity. The vibration velocity scales with the frequency.

For very small vibration amplitude ($\hat{s} \ll \lambda$) the contributions from the Bessel function can be approximated with

$$\mathrm{J}_0(a) \approx 1 \tag{SI.3}$$

$$\mathrm{J}_1(a) = -\left[\mathrm{J}_{-1}(a)\right] \approx \frac{a}{2}. \tag{SI.4}$$

This approximation leads to equation (15) of the main document.

2 Solution of the Integrals

Due to the components of the phase factor Φ, the numerator integral in equation (20) of the main document can be split into two integrals (substituted by Υ_1 and Υ_2) and the denominator integral Ψ

$$\frac{\hat{s}_\mathrm{est}}{\hat{s}} \approx \frac{\Re\left\{\Upsilon_1+\Upsilon_2\right\}}{\Psi}. \tag{SI.5}$$

The first numerator integral Υ_1 is

$$\Upsilon_1 = \left(1-\frac{\lambda}{2\pi}\frac{1}{z_\mathrm{R}}\right)\iint\limits_{A_\mathrm{D}} E_\mathrm{m}^\mathrm{W}(\rho')\,E_\mathrm{r}^\mathrm{W}(\rho')\,\exp\left(-\mathrm{j}\,2\pi\frac{x'}{\Lambda\beta}\right)\mathrm{d}A'. \tag{SI.6}$$

For better understanding, z_R remains the Rayleigh range after focusing with the microscope objective. Thus, the second numerator integral Υ_2 is

$$\Upsilon_2 = \frac{1}{2\beta^2 z_\mathrm{R}^2}\iint\limits_{A_\mathrm{D}} E_\mathrm{m}^\mathrm{W}(\rho')\,E_\mathrm{r}^\mathrm{W}(\rho')\,\rho'^2\,\exp\left(-\mathrm{j}\,2\pi\frac{x'}{\Lambda\beta}\right)\mathrm{d}A'. \tag{SI.7}$$

The denominator integral Ψ is

$$\Psi = \iint_{A_\mathrm{D}} E_\mathrm{m}^\mathrm{W}\left(\rho'\right) E_\mathrm{r}^\mathrm{W}\left(\rho'\right) \mathrm{d}A' \,. \tag{SI.8}$$

For the analytic solution of these integrals it is assumed that the beams are perfectly matched, so that the beam waist radii of the reference and the measurement beam at the interference are identical. The common beam waist on the photodetector is w_D. Further, it is assumed that the sensitive are of the detector is larger than this waist radius. Thus, the integration over the sensitive area A_D can be approximated with the integration from $-\infty$ to ∞. A smaller area of the photodetector in respect to the beam waist results in a spatial filtering which has an effect lateral resolution comparable to confocalization in microscopy[2].

For the analytical solution of the integrals, the following known solutions of definite integrals[1] are utilized

$$\int_{-\infty}^{\infty} \exp\left(-2\frac{x^2}{a^2}\right) \mathrm{d}x = \sqrt{\frac{\pi}{2}}\,a \qquad (\text{for } a>0) \tag{SI.9}$$

$$\iint_{-\infty}^{\infty} \exp\left(-2\frac{\rho^2}{a^2}\right) \mathrm{d}A = \frac{\pi}{2}\,a^2 \tag{SI.10}$$

$$\int_{-\infty}^{\infty} x^2 \exp\left(-2\frac{x^2}{a^2}\right) \mathrm{d}x = \sqrt{\frac{\pi}{2}}\,\frac{a^3}{4} \qquad (\text{for } a>0)\,. \tag{SI.11}$$

And the Fourier transformations[1] with the angular frequency ω

$$\int_{-\infty}^{\infty} \exp\left(-2\frac{x^2}{a^2}\right) \exp\left(-\mathrm{j}\,\omega x\right) \mathrm{d}x = \sqrt{\frac{\pi}{2}}\,a \exp\left(-\frac{\omega^2 a^2}{8}\right) \tag{SI.12}$$

$$\int_{-\infty}^{\infty} x^2 \exp\left(-2\frac{x^2}{a^2}\right) \exp\left(-\mathrm{j}\,\omega x\right) \mathrm{d}x = \frac{4-\omega^2 a^2}{16}\sqrt{\frac{\pi}{2}}\,a^3 \exp\left(-\frac{\omega^2 a^2}{8}\right)\,. \tag{SI.13}$$

The solution of denominator integral Ψ (with the spot profile in equation (3) is found with equation (SI.10) to be

$$\Psi \approx \iint_{-\infty}^{\infty} E_\mathrm{m}^\mathrm{W}\left(\rho'\right) E_\mathrm{r}^\mathrm{W}\left(\rho'\right) \mathrm{d}A' = \hat{E}_\mathrm{m}\,\hat{E}_\mathrm{r} \iint_{-\infty}^{\infty} \exp\left(-2\frac{\rho'^2}{w_\mathrm{D}^2}\right) \mathrm{d}A' = \hat{E}_\mathrm{m}\,\hat{E}_\mathrm{r}\,\frac{\pi}{2}\,w_\mathrm{D}^2\,. \tag{SI.14}$$

The first numerator integral Υ_1 is (with the spot profile in equation (3), $\mathrm{d}A = \mathrm{d}x'\,\mathrm{d}y'$, and $\rho'^2 = x'^2 + y'^2$)

$$\Upsilon_1 \approx \hat{E}_\mathrm{m}\,\hat{E}_\mathrm{r}\left(1-\frac{\lambda}{2\pi}\frac{1}{z_\mathrm{R}}\right) \int_{-\infty}^{\infty} \exp\left(-2\frac{y'^2}{w_\mathrm{D}^2}\right) \mathrm{d}y' \int_{-\infty}^{\infty} \exp\left(-2\frac{x'^2}{w_\mathrm{D}^2}\right) \exp\left(-\mathrm{j}\,2\pi\frac{x'}{\Lambda\beta}\right) \mathrm{d}x'\,. \tag{SI.15}$$

With the solution of the integrals in equation (SI.9) and equation (SI.12) it is

$$\Upsilon_1 \approx \hat{E}_\mathrm{m}\,\hat{E}_\mathrm{r}\left(1-\frac{\lambda}{2\pi}\frac{1}{z_\mathrm{R}}\right) \sqrt{\frac{\pi}{2}}\,w_\mathrm{D} \times \sqrt{\frac{\pi}{2}}\,w_\mathrm{D} \exp\left(-\frac{4\pi^2 w_\mathrm{D}^2}{8\,\Lambda^2 \beta^2}\right)\,, \tag{SI.16}$$

yielding after concentration

$$\Upsilon_1 \approx \hat{E}_\mathrm{m}\,\hat{E}_\mathrm{r}\left(1-\frac{\lambda}{2\pi}\frac{1}{z_\mathrm{R}}\right) \frac{\pi}{2}\,w_\mathrm{D}^2 \exp\left(-\frac{\pi^2 w_\mathrm{D}^2}{2\,\Lambda^2 \beta^2}\right)\,. \tag{SI.17}$$

With the spot profile in equation (3) and $\rho'^2 = x'^2 + y'^2$, the second numerator integral Υ_2 in equation (SI.7) is

$$\Upsilon_2 \approx \frac{\hat{E}_\mathrm{m}\,\hat{E}_\mathrm{r}}{2\,\beta^2 z_\mathrm{R}^2} \iint_{-\infty}^{\infty} \exp\left(-2\frac{x'^2+y'^2}{w_\mathrm{D}^2}\right) \left(x'^2+y'^2\right) \exp\left(-\mathrm{j}\,2\pi\frac{x'}{\Lambda\beta}\right) \mathrm{d}x'\,\mathrm{d}y'\,. \tag{SI.18}$$

Utilizing the distributive law the second numerator integral Υ_2 is split into two independent integrals

$$\begin{aligned}\Upsilon_2 \approx \frac{\hat{E}_\mathrm{m}\,\hat{E}_\mathrm{r}}{2\,\beta^2\,z_\mathrm{R}^2}\Bigg[&\iint\limits_{-\infty}^{\infty} x'^2 \exp\left(-2\,\frac{x'^2+y'^2}{w_\mathrm{D}^2}\right)\exp\left(-\mathrm{j}\,2\pi\frac{x'}{\Lambda\beta}\right)\mathrm{d}x'\,\mathrm{d}y'\\ &+\iint\limits_{-\infty}^{\infty} y'^2 \exp\left(-2\,\frac{x'^2+y'^2}{w_\mathrm{D}^2}\right)\exp\left(-\mathrm{j}\,2\pi\frac{x'}{\Lambda\beta}\right)\mathrm{d}x'\,\mathrm{d}y'\Bigg]\\ =\frac{\hat{E}_\mathrm{m}\,\hat{E}_\mathrm{r}}{2\,\beta^2\,z_\mathrm{R}^2}\Bigg[&\int\limits_{-\infty}^{\infty}\exp\left(-2\,\frac{y'^2}{w_\mathrm{D}^2}\right)\mathrm{d}y'\times\int\limits_{-\infty}^{\infty} x'^2\exp\left(-2\,\frac{x'^2}{w_\mathrm{D}^2}\right)\exp\left(-\mathrm{j}\,2\pi\frac{x'}{\Lambda\beta}\right)\mathrm{d}x'\\ &+\int\limits_{-\infty}^{\infty} y'^2\exp\left(-2\,\frac{y'^2}{w_\mathrm{D}^2}\right)\mathrm{d}y'\times\int\limits_{-\infty}^{\infty}\exp\left(-2\,\frac{x'^2}{w_\mathrm{D}^2}\right)\exp\left(-\mathrm{j}\,2\pi\frac{x'}{\Lambda\beta}\right)\mathrm{d}x'\Bigg]. \end{aligned}\tag{SI.19}$$

With the solutions of the separate integrals in equations (SI.9), (SI.13), (SI.11), and (SI.12) from the literature, one yields

$$\Upsilon_2 \approx \frac{\hat{E}_\mathrm{m}\,\hat{E}_\mathrm{r}}{2\,\beta^2\,z_\mathrm{R}^2}\left[\sqrt{\frac{\pi}{2}}\,w_\mathrm{D}\,\frac{4-\dfrac{4\pi^2\,w_\mathrm{D}^2}{\Lambda^2\,\beta^2}}{16}\sqrt{\frac{\pi}{2}}\,w_\mathrm{D}^3\exp\left(-\frac{\pi^2\,w_\mathrm{D}^2}{2\Lambda^2\,\beta^2}\right)+\sqrt{\frac{\pi}{2}}\,\frac{w_\mathrm{D}^3}{4}\sqrt{\frac{\pi}{2}}\,w_\mathrm{D}\exp\left(-\frac{\pi^2\,w_\mathrm{D}^2}{2\Lambda^2\,\beta^2}\right)\right],\tag{SI.20}$$

which concentrated becomes

$$\Upsilon_2 \approx \frac{\hat{E}_\mathrm{m}\,\hat{E}_\mathrm{r}}{2\,\beta^2\,z_\mathrm{R}^2}\,\frac{\pi}{2}\,\frac{w_\mathrm{D}^4}{4}\left(2-\frac{\pi^2\,w_\mathrm{D}^2}{\Lambda^2\,\beta^2}\right)\exp\left(-\frac{\pi^2\,w_\mathrm{D}^2}{2\Lambda^2\,\beta^2}\right).\tag{SI.21}$$

The resubstitution of the numerator integral Υ_1 from equation (SI.17), numerator integral Υ_2 from equation (SI.21), and the denominator integral Ψ from equation (SI.14) into equation (SI.5) gives

$$\begin{aligned}\frac{\hat{s}_\mathrm{est}}{\hat{s}} &\approx \left(1-\frac{\lambda}{2\pi\,z_\mathrm{R}}\right)\exp\left(-\frac{\pi^2\,w_\mathrm{D}^2}{2\Lambda^2\,\beta^2}\right)+\frac{1}{2\,\beta^2\,z_\mathrm{R}^2}\,\frac{w_\mathrm{D}^2}{4}\left(2-\frac{\pi^2\,w_\mathrm{D}^2}{\Lambda^2\,\beta^2}\right)\exp\left(-\frac{\pi^2\,w_\mathrm{D}^2}{2\Lambda^2\,\beta^2}\right)\\ &=\left[1-\frac{\lambda}{2\pi\,z_\mathrm{R}}+\frac{w_\mathrm{D}^2}{8\,\beta^2\,z_\mathrm{R}^2}\left(2-\frac{\pi^2\,w_\mathrm{D}^2}{\Lambda^2\,\beta^2}\right)\right]\exp\left(-\frac{\pi^2\,w_\mathrm{D}^2}{2\Lambda^2\,\beta^2}\right).\end{aligned}\tag{SI.22}$$

Using the definition of the Rayleigh range z_R (at the waist) in equation (9) and with the original waist radius $w_0 = w_\mathrm{D}/\beta$ at the specimen, one yields

$$\begin{aligned}\frac{\hat{s}_\mathrm{est}}{\hat{s}} &\approx \left[1-\frac{\lambda}{2\pi\,z_\mathrm{R}}+\frac{\lambda}{8\pi\,z_\mathrm{R}}\left(2-\frac{\pi\lambda\,z_\mathrm{R}}{\Lambda^2}\right)\right]\exp\left(-\frac{\pi\,\lambda\,z_\mathrm{R}}{2\Lambda^2}\right)\\ &=\left(1-\frac{\lambda}{2\pi\,z_\mathrm{R}}+\frac{\lambda}{4\pi\,z_\mathrm{R}}-\frac{\lambda}{8\pi\,z_\mathrm{R}}\,\frac{\pi\,\lambda\,z_\mathrm{R}}{\Lambda^2}\right)\exp\left(-\frac{\pi\,\lambda\,z_\mathrm{R}}{2\Lambda^2}\right)\\ &=\left(1-\frac{\lambda}{4\pi\,z_\mathrm{R}}-\frac{\lambda^2}{8\Lambda^2}\right)\exp\left(-\frac{\pi\,\lambda\,z_\mathrm{R}}{2\Lambda^2}\right).\end{aligned}\tag{SI.23}$$

Replacing the Rayeigh range with the beam divergence $z_\mathrm{R} = \lambda/\left[\pi\,\tan^2\left(\theta_\mathrm{div}\right)\right]$ gives

$$\frac{\hat{s}_\mathrm{est}}{\hat{s}} \approx \left[1-\frac{\tan^2\left(\theta_\mathrm{div}\right)}{4}-\frac{\lambda^2}{8\Lambda^2}\right]\exp\left[-\frac{\lambda^2}{2\Lambda^2}\,\frac{1}{\tan^2\left(\theta_\mathrm{div}\right)}\right].\tag{SI.24}$$

In microscopy a common parameter of a microscope objective is the numerical aperture NA (in air) which can be approximated by the beam divergence θ_div as

$$\mathrm{NA} = \sin\left(\theta_\mathrm{div}\right) \approx \tan\left(\theta_\mathrm{div}\right).\tag{SI.25}$$

Note that due to the approximation of paraxial beam propagation this is only valid for moderate NAs. With this equation the remaining dependencies of the systematic vibration-amplitude deviation are on the ratio Λ/λ and the NA the equation (21) can be derived

3 Details on the specimen

The experimental validation was conducted with a two-port front-end filter (Epcos B3530) typically utilized for communication applications which provides a nominal central frequency at 433.92 MHz with a bandwidth of 0.95 MHz. The cap of the metal package (TO39) has been removed to provide the necessary optical access to the SAW. During the experiment, a vector network analyzer (S5065 from Copper Mountain Technology, Inc.) excited with an input power of +5 dBm at the single frequency of 433.35 MHz and analyzed the scattering parameters. Due to the removal of the hermetic sealing, the insertion attenuation of the SAW filter has been increased by 10 dB (2.3 dB are specified) whereas the transmission characteristics remained similar.

A single location of the laser spot on the central, reflective shielding between the inter-digital transducers for all measurements of the experiment was chosen which provides a sufficient and invariable amplitude. Everything else is not decisive for the meaningfulness of our experiment.

4 Determination of uncertainties in the experiment

For the validation of the approximation equation (22) from the model, the uncertainties of each measurement in NA and relative measured vibration amplitude $\hat{s}_{\text{est}}/\hat{s}$ were determined by Gaussian propagation of the uncertainties of the input parameters according to the guide to the expression of uncertainty in measurement (GUM)[3]. All uncertainties are given with a confidence level of 68.3%.

For the determination of the NA, the beam diameter at the entrance pupil was measured with a CMOS camera (Basler acA1920-40ua) and a MATLAB evaluation. The calculation followed equation (SI.25). Therefore, the input parameters are the pixel size, determination of the beam diameter and the focal length of the microscope objective. The uncertainty of the beam diameter was determined statistically to 29 pixels over a sample of measurements (according to method A). The uncertainties of the nominal pixel size and the nominal focal length of the microscope objective have been estimated to 5% and 2% respectively (according to method B).

For the determination of the relative measured vibration amplitude, the uncertainty of the measured vibration amplitude $\hat{s}_{\text{est}}$ was determined statistically over 10 successive vibration measurements (at static measurement conditions) (according to method A). The resulting uncertainty for the measured vibration amplitude was less than 20 pm. The real vibration amplitude $\hat{s}$ was estimated by a least-mean-squares fit (in MATLAB) with the approximation equation (22) to be 175 pm with an uncertainty of 5%.

For each input parameter the local sensitivity was determined. Thus, a resulting uncertainty for the NA and the relative measured vibration amplitude of each measurement point in Figure 4 in the main document has been quantified and plotted.

The input parameter Λ/λ for the approximation equation (22) was determined in two experiments. The SAW wavelength Λ was measured by the filter pitch 7.3, μm via a microscopic measurement (after a calibration with USAF target). The wavelength λ of the laser was determined with a wavemeter (HighFinesse WS6-600). The uncertainties for these measurements were negletable against the other uncertainties.

References

1. Bronstein, I. N. & Semendjajew, K. A. *Taschenbuch der Mathematik* (Deutsch, Thun, 2001), 5 edn.
2. Corle, T. R. & Kino, G. S. *Confocal scanning optical microscopy and related imaging systems* (Academic Press, San Diego, 1996).
3. Joint Committee for Guides in Metrology. JCGM 100: Evaluation of measurement data - guide to the expression of uncertainty in measurement. Tech. Rep., JCGM (2008).
